# Antimicrobial susceptibility of *Campylobacter jejuni* and *Campylobacter coli*: comparison between Etest and a broth dilution method

**DOI:** 10.1186/s12941-018-0275-8

**Published:** 2018-05-23

**Authors:** Maya Azrad, Linda Tkhawkho, Natalia Isakovich, Orna Nitzan, Avi Peretz

**Affiliations:** 1 Clinical Microbiology Laboratory, Baruch Padeh Medical Center, Poriya, Hanna Senesh 818/2, Tiberias, Israel; 2 The Azrieli Faculty of Medicine, Bar Ilan University, Galilee, Israel; 3 Unit of Infectious Diseases, Baruch Padeh Medical Center, Poriya, Tiberias, Israel

**Keywords:** Etest, Sensititre kit, *Campylobacter jejuni*, *Campylobacter coli*

## Abstract

**Background:**

*Campylobacter* is a leading cause of foodborne gasteroenteritis worldwide. Antimicrobial susceptibility testing for *Campylobacter* spp. is not routinely performed by most clinical laboratories. However, the emergence of resistant isolates strengthens the importance of antimicrobial susceptibility testing and the critical need for epidemiologic surveillance. The aim of this study was to compare the efficacy of Etest and Sensititre kit (a broth microdilution method) as methods for susceptibility tests and the critical need for epidemiologic surveillance. The aim of this study was to compare the efficacy of Etest and Sensititre kit (a broth microdilution method) as methods for susceptibility testing of *Campylobacter* spp. to tetracycline, erythromycin, and ciprofloxacin.

**Methods:**

Sixty-six *Campylobacter* isolates were collected from feces samples and subjected to susceptibility testing by Etest and Sensititre, a broth microdilution kit for tetracycline, erythromycin, and ciprofloxacin. Minimal inhibitory concentration (MIC) results of each method were determined and compared.

**Results:**

Similar MIC interpretations for tetracycline, erythromycin, and ciprofloxacin were found in 97%, 98.5%, and 100% of the isolates, respectively, indicating a good level of agreement between Etest and Sensititre (*p* < 0.0001); additionally, the correlation between the two methods was highly significant for the three tested antibiotics (*p* < 0.0001).

**Conclusions:**

Both the broth microdilution and the Etest are reliable and convenient methods for testing antimicrobial susceptibility of *Campylobacter* spp. The Sensititre kit has the advantages of high availability and the automation.

## Background

*Campylobacter* is a leading cause of foodborne gastroenteritis worldwide [1]. In 2015, the Foodborne Diseases Active Surveillance Network (FoodNet) reported an incidence rate of 12.82 per 100,000 persons [2]. The most prevalent species responsible for human infections are *Campylobacter jejuni* and *Campylobacter coli* [1, 3–5]. Usually, *Campylobacter* infections result in a self-limited watery diarrhea, accompanied by fever and abdominal cramps [6]. However, severe and prolonged illness has also been reported [1]. Extra-gastrointestinal infections include bacteremia, reactive arthritis, meningitis, lung infections, and brain abscesses. Additionally, several diseases and gastrointestinal conditions were associated with campylobacteriosis including Guillain–Barré syndrome, Miller Fisher syndrome, inflammatory bowel diseases, Barrett’s esophagus, and colorectal cancer [6]. Antibiotic treatment is warranted in immuno suppressed patients and in severe cases [3, 4]. The drug of choice is macrolides such as erythromycin and clarithromycin. Fluoroquinolones (e.g., ciprofloxacin) are also commonly used in empirical treatment of undiagnosed diarrhea. Additional prescribed drugs are tetracycline, doxycycline, and chloramphenicol [1].

Campylobacteriosis incidence is globally rising [6]. In Israel, which is characterized by a high *Campylobacter* morbidity, there is a requirement to report *Campylobacter* infections to the Ministry of Health [7]. While in 2010 the incidence of *Campylobacter* infections in the USA was 12.3 per 100,000 population [8], in Israel, the incidence has increased 2.93-fold since 1999 (from 31.04 to 90.99 cases per 100,000 population) [7].

Antimicrobial susceptibility testing (AST) for *Campylobacter* spp. is not routinely performed by most clinical laboratories. However, the emergence of resistant isolates strengthens the importance of AST and the critical need for epidemiologic surveillance [1]. For example, in the USA, in 2015 2.7 and 12.7% of *C. jejuni* and *C. coli*, respectively, were resistant to erythromycin. Resistance rates to ciprofloxacin were 25.3 and 39.8%, respectively, for *C. jejuni* and *C. coli* isolates [9].

Several methods for in vitro AST of *Campylobacter* spp. are currently available, including disk diffusion, Etest^®^, and dilution methods (broth and agar dilution) [1]. Currently the acceptable methods are agar and broth microdilution in which a microorganism suspension is inoculated with serial dilutions of an antibiotic agent and the minimum inhibitory concentration (MIC) is determined following incubation [1]. As this technique is inconvenient and time-consuming, commercial kits such as the Sensititre^®^ (Trek Diagnostic Systems, UK) have been developed to facilitate the susceptibility testing. Here we present a comparison of the MIC results, interpreted according to The European Committee on Antimicrobial Susceptibility Testing (EUCAST) guidelines, of 66 *Campylobacter* isolates from feces cultures for erythromycin, ciprofloxacin, and tetracycline between Etest^®^ and the Sensititre^®^ broth microdilution plate.

## Methods

### Sample collection

The study was performed at the clinical microbiology laboratory of the Baruch Padeh Medical Center, Poriya, in northern Israel. From May 2015 to February 2017, a total of 66 *Campylobacter* isolates were collected from feces samples of patients admitted to the hospital and diagnosed as suffering from Campylobacteriosis. Forty-four of the patients were children and 22 were adults. All feces samples were routinely subcultured on *Campylobacter* selective agar (BD Diagnostics, Sparks, MD) and incubated at 42 °C for 48 h with CampyGen™ (Thermo Fischer Scientific, MA, USA) sachet for the generation of microaerophilic conditions.

### *Campylobacter* isolates

Following incubation of feces samples, *Campylobacter* colonies were identified according to morphologic and biochemical characteristics. Final identification was performed by MALDI-TOF Mass Spectrometry System (Bruker Daltonics, Bremen, Germany). All isolates, 50 *C. jejuni* and 16 *C. coli*, were sent to a reference laboratory for final confirmation of the species.

### In vitro antibiotics susceptibility tests (AST)

All *Campylobacter* isolates were grown for 24 h at 37 °C in microaerophilic conditions before conducting susceptibility tests. *C. jejuni* ATCC 33560 was used for quality control.

### Etest

After 48 h, several colonies were suspended in Brain Heart Infusion (BHI) broth to a turbidity of 0.5 McFarland. The suspension was seeded on three Mueller–Hinton agar plates containing lysed horse blood (Hy Laboratories Ltd., Rehovot, Israel) and then one Etest strip (bioMérieux, Durham, NC) was put on each agar plate for erythromycin, ciprofloxacin, and tetracycline. Plates were incubated at 37 °C for 48 h in microaerophilic conditions. MIC values were determined after 48 h according to EUCAST guidelines: resistance to tetracycline is defined at MIC values above 2 mg/L; resistance to ciprofloxacin is defined at MIC values above 0.5 mg/L; resistance to erythromycin is defined at MIC values above 4 mg/L for *C. jejuni* and 8 mg/L for *C. coli*.

### Sensititre^®^ broth microdilution plate

We performed broth microdilution (BMD) susceptibility test using the Sensititre^®^ broth microdilution plate, according to the manufacturer’s instructions. Briefly, each plate was in a dried form and contained a standard panel of serial dilutions of antimicrobial agents. Isolated colonies were suspended in 5 mL cation-adjusted Mueller–Hinton broth with TES buffer (CAMHBT) and turbidity was adjusted to 0.5 McF. The suspension (100 µL) was transferred into 11 mL cation-adjusted Mueller–Hinton broth with TES buffer and lysed horse blood (CAMHBT + LHB) and mixed. The new suspension (100 µL) was transferred to each well, which contained different concentrations of the antibiotic agent. The plate was sealed and incubated at 37 °C for 48 h in microaerophilic conditions. Following incubation, plates were visually read, searching for turbidity as an indicator of microbial growth. MIC values were determined as the lowest antibiotic concentration that inhibited microbial growth. Resistance to the antibiotic agent was determined according to EUCAST guidelines, as mentioned above. Each batch of BMD tests included *Campylobacter* control strains. The limits of MIC for *C. jejuni* ATCC 33560 were adopted from the manufacturer’s instructions: for erythromycin, 0.5–2.0 µg/mL; for ciprofloxacin, 0.06–0.25 µg/mL; and for tetracycline, 0.25–2 µg/mL.

### Statistical analysis

For calculation of categorical agreement between the two methods, the percentage of agreement was calculated as the percentage of isolates that had the same result interpretation (sensitive or resistant) by the two methods. The level of categorical agreement was analyzed by calculating Kappa coefficients, with the acceptable following values: r < 0 poor agreement; 0 ≤ r ≤ 0.2 slight agreement; 0.21 ≤ r ≤ 0.4 fair agreement; 0.41 ≤ r ≤ 0.6 moderate agreement; 0.61 ≤ r ≤ 0.8 substantial agreement, and 0.81 ≤ r ≤ 1 almost perfect agreement.

The very major error rate was defined as the percentage of isolates that had a “susceptible” result by Etest and a “resistant” result by BMD. The major error rate was defined as the percentage of isolates that had a “resistant” result by Etest and a “susceptible” result by BMD.

Pearson correlation coefficients were calculated to test the level of agreement. Pearson coefficient range is between − 1 and 1, where zero means no correlation, 0 ≤ r ≤ 1 reflects the strength of a positive correlation, and − 1 ≤ r ≤ 0 reflects the strength of a negative correlation. The higher the coefficient, the stronger the correlation.

Statistical significance was determined with *p* < 0.05. The data was analyzed using the SPSS^®^ version 23.

## Results

Sixty-six *Campylobacter* isolates were collected in the current study; of these, 50 *Campylobacter jejuni* and 16 *Campylobacter coli* were identified.

In comparison of Etest with Sensititre for MIC determination, categorical agreement for tetracycline, erythromycin, and ciprofloxacin were 97% (64/66), 98.5% (65/66), and 100% (66/66), respectively (Table 1). Three isolates, all of which were *C. jejuni*, were interpreted as sensitive according to Etest and resistant according to Sensititre (one to erythromycin and two to tetracycline). Thus, the very major error rates were 3, 2.5, and 0% for tetracycline, erythromycin, and ciprofloxacin, respectively. No minor or major errors were observed. Kappa coefficients of 0.653, 0.66, and 1 (*p* < 0.0001) were calculated for tetracycline, erythromycin, and ciprofloxacin, respectively, indicating a substantial level of agreement for tetracycline and erythromycin, and an almost perfect agreement for ciprofloxacin (Table 1).

**Table 1:** Analysis of correlation and categorical agreement levels between Etest and Sensititre kit (broth microdilution method) as methods for susceptibility testing of *Campylobacter* isolates to tetracycline, erythromycin, and ciprofloxacin, using Sensititre and Etest methods

Antibiotic agent	Percentage of categorical agreement	Kappa coefficient r (*p* value)	Pearson correlation coefficient (*p* value)
Tetracycline	97	0.653 (< 0.0001)	0.645 (< 0.0001)
Erythromycin	98.5	0.66 (< 0.0001)	0.588 (< 0.0001)
Ciprofloxacin	100	1 (< 0.0001)	0.865 (< 0.0001)

Additionally, the correlation between methods following logarithmic transformation was good and highly significant for all the tested antibiotics (*p* < 0.0001), with Pearson correlation coefficients of 0.645, 0.588, and 0.865 for tetracycline, erythromycin, and ciprofloxacin, respectively.

According to EUCAST criteria, 97% (64/66) and 94% (62/66) of our isolates were resistant to tetracycline using Sensititre and Etest, respectively (Table 2). Resistance rates of *C. jejuni* to tetracycline were 100% (50/50) and 96% (48/50), using Sensititre and Etest, respectively. Resistance rates of *C. coli* to tetracycline were 87.5% (14/16), using both methods.

**Table 2:** Resistance rates of *Campylobacter* isolates to tetracycline, erythromycin, and ciprofloxacin, using Sensititre and Etest methods

Species	Antibiotic agent					
	Tetracycline		Erythromycin		Ciprofloxacin	
	Sensititre	Etest	Sensititre	Etest	Sensititre	Etest
*C. jejuni* (n = 50)	100	96	4	2	96	96
*C. coli* (n = 16)	87.5	87.5	0	0	93.75	93.75
Total (n = 66)	97	94	3.1	1.5	95.45	95.45

Erythromycin resistance rates, according to EUCAST guidelines, were 3.03% (2/66) and 1.5% (1/66), as determined by Sensititre and Etest, respectively. Resistance rates of *C. jejuni* to erythromycin were 4% (2/50) and 2% (1/50), using Sensititre and Etest, respectively. All *C. coli* isolates were sensitive to erythromycin.

Resistance rates to ciprofloxacin were high for both species and similar using both methods; overall, 95.45% (63/66) of the isolates were resistant to ciprofloxacin; 96% (48/50) of the *C. jejuni* isolates and 93.75 (15/16) of the *C. coli* isolates were resistant to ciprofloxacin (Table 2).

## Discussion

*Campylobacter* resistance to antibiotics is on rise worldwide, emphasizing the importance of AST performance [1, 5, 10]. In this study, a comparison of Sensititre kit (broth microdilution method) and Etest for MIC determination for three antibiotics revealed excellent concordance between these methods. This result is similar to previous studies that compared the Etest and a broth microdilution method [3, 4]. We found a highly significant correlation between the Sensititre kit and the Etest method for the tested antibiotics (*p* < 0.0001). Additionally, no major errors were found and the very major error rates were low (0–3%). The Etest method generally produced higher MIC values, a phenomenon that was previously described [4]. Both the broth microdilution and the Etest methods are reliable, rapid, and easy-to-perform techniques. The Sensititre kit is advantageous due to its availability and the optional automated reading of the plates.

The considerably low resistance rate to erythromycin (1.5–3.1%) in the current study is lower than that found in other countries such as in Bulgaria (31%), Singapore (51%), and Ghana (96%) [5, 11]. In the USA and Canada, the prevalence of resistant isolates was lower than 10% [10–16]; while 2–4% of *C. jejuni* and 0% of *C. coli* strains were resistant to erythromycin (Table 2), the reported rates in Israel in 2014 were 1 and 8% for *C. jejuni* and *C. coli*, respectively (unpublished data). Our results may point to wise/careful use of antimicrobial treatment in campylobacteriosis. In the current study, 95.45% of the isolates (96% of *C. jejuni* and 93.75% of *C. coli*) were resistant to ciprofloxacin. This extremely high resistance rate is much higher than the reported resistance levels in the USA and Canada, in which 19–47% of human *Campylobacter* isolates were resistant to ciprofloxacin [10]. In comparison, the prevalence of fluoroquinolones-resistant *Campylobacter* in Thailand and Hong Kong was found to be above 80% [10, 17, 18], 35% in Ghana [5], and 17–99% in Europe, with the highest resistance rate in Spain [10].

However, our result is similar to the resistance levels found in 2014 by the Israel Ministry of Health that reported 93 and 98% resistance rates among *C. jejuni* and *C. coli* strains, respectively (unpublished data). Additionally, ciprofloxacin resistance has increased over the years [4, 19]; in Israel, for example, in 2009 resistance rates were 85 and 80% for *C. jejuni* and *C. coli*, respectively (unpublished data).

We found a resistance rate of 94–97% (Etest/Sensititre) to tetracycline among our strains. Interestingly, our resistance rate of *C. jejuni* was 96–100%, much higher than the reported rate in Israel in 2014, which was 53%. These differences may be explained by demographic characteristics of the study area, northern Galilee, with a rural population having close contact with animals. As *Campylobacter* infection is a zoonotic disease, resistance to antibiotics is not only influenced by antibiotics use in human but also in animals [10]. A similar high rate was also observed in Ghana (92%) [5]. Lower resistance rates were measured in Spain (72%) and in Ethiopia (22%) [5, 20, 21]. Resistance to tetracycline is apparently an outcome of the antimicrobial use in veterinary husbandry and in food production [5]. Overall, the increasing resistance among *Campylobacter* strains underscores the need to curb antibiotic use in the veterinary domain and in humans.

In summary, Etest and Sensititre kit can be easily adapted for monitoring susceptibility of *Campylobacter* spp. Obtaining data regarding prevalence and distribution of resistant strains is very important, not only for therapy adjustment but also for epidemiologic surveillance, especially in countries such as Israel, which is characterized by a high prevalence of campylobacteriosis.

## References

[1] Ge B, Wang F, Sjölund-Karlsson M, McDermott PF (2013). Antimicrobial resistance in Campylobacter: susceptibility testing methods and resistance trends. J Microbiol Methods.

[2] Department of Health U, Services H, for Disease Control C, Center for Emerging N, Infectious Diseases Z. Foodborne disease active surveillance network 2015. Surveillance Report (Final Data).

[3] Luber P, Bartelt E, Genschow E, Wagner J, Hahn H (2003). Comparison of broth microdilution, E Test, and Agar dilution methods for antibiotic susceptibility testing of *Campylobacter jejuni* and *Campylobacter coli* comparison of broth microdilution, E Test, and Agar dilution methods for antibiotic susceptibilit. J Clin Microbiol.

[4] Van der Beek MT, Claas ECJ, Mevius DJ, van Pelt W, Wagenaar JA, Kuijper EJ (2010). Inaccuracy of routine susceptibility tests for detection of erythromycin resistance of *Campylobacter jejuni* and *Campylobacter coli*. Clin Microbiol Infect.

[5] Karikari AB, Obiri-Danso K, Frimpong EH, Krogfelt KA (2017). Antibiotic resistance in Campylobacter isolated from patients with gastroenteritis in a teaching hospital in Ghana. Open J Med Microbiol..

[6] Kaakoush NO, Castaño-Rodríguez N, Mitchell HM, Man SM (2015). Global epidemiology of Campylobacter infection. Clin Microbiol Rev.

[7] Weinberger M, Lerner L, Valinsky L, Moran-Gilad J, Nissan I, Agmon V (2013). Increased incidence of *Campylobacter* spp. infection and high rates among children, Israel. Emerg Infect Dis.

[8] Crim S, Henao O, Huang J, Luna R, Mahon B, Mody R, et al. Foodborne Active Disease Surveillance Network 2010.

[9] NARMS Now. 2017. https://wwwn.cdc.gov/narmsnow/. Accessed 16 July 2017.

[10] Luangtongkum T, Jeon B, Han J, Plummer P, Logue CM, Zhang Q (2009). Antibiotic resistance in Campylobacter: emergence, transmission and persistence. Future Microbiol..

[11] Gibreel A, Taylor DE (2006). Macrolide resistance in *Campylobacter jejuni* and *Campylobacter coli*. J Antimicrob Chemother.

[12] Belanger AE, Shryock TR (2007). Macrolide-resistant Campylobacter: the meat of the matter. J Antimicrob Chemother.

[13] Englen MD, Hill AE, Dargatz DA, Ladely SR, Fedorka-Cray PJ (2007). Prevalence and antimicrobial resistance of Campylobacter in US dairy cattle. J Appl Microbiol.

[14] Guévremont E, Nadeau E, Sirois M, Quessy S (2006). Antimicrobial susceptibilities of thermophilic Campylobacter from humans, swine, and chicken broilers. Can J Vet Res.

[15] Lé Vesque S, Frost E, Michaud S (2007). Comparison of antimicrobial resistance of *Campylobacter jejuni* isolated from humans, chickens, raw milk, and environmental water in Qué bec. J Food Prot.

[16] Luangtongkum T, Morishita TY, Ison AJ, Huang S, McDermott PF, Zhang Q (2006). Effect of conventional and organic production practices on the prevalence and antimicrobial resistance of *Campylobacter* spp. in poultry. Appl Environ Microbiol.

[17] Chu Y-W, Chu M-Y, Luey K-Y, Ngan Y-W, Tsang K-L, Kam K-M (2004). Genetic relatedness and quinolone resistance of *Campylobacter jejuni* strains isolated in 2002 in Hong Kong. J Clin Microbiol.

[18] Isenbarger DW, Hoge CW, Srijan A, Pitarangsi C, Vithayasai N, Bodhidatta L (2002). Comparative antibiotic resistance of diarrheal pathogens from Vietnam and Thailand, 1996–1999. Emerg Infect Dis.

[19] Engberg J, Aarestrup FM, Taylor DE, Gerner-Smidt P, Nachamkin I (2001). Quinolone and macrolide resistance in *Campylobacter jejuni* and *C. coli*: resistance mechanisms and trends in human isolates. Emerg Infect Dis.

[20] Ewnetu D, Mihret A (2010). Prevalence and antimicrobial resistance of *Campylobacter* isolates from humans and chickens in Bahir Dar, Ethiopia. Foodborne Pathog Dis..

[21] Prats G, Mirelis B, Llovet T, Muñoz C, Miró E, Navarro F (2000). Antibiotic resistance trends in enteropathogenic bacteria isolated in 1985–1987 and 1995–1998 in Barcelona. Antimicrob Agents Chemother.

